# Prevalence and Genetic Characterization of *Giardia duodenalis* and *Blastocystis* sp. in Horses in Shanxi Province, North China

**DOI:** 10.3390/ani16121845

**Published:** 2026-06-15

**Authors:** Xun-Zhi Liu, Nan Su, Wen Li, Dong-Yang Wang, Ze-Dong Zhang, Xing-Quan Zhu, Wen-Wei Gao

**Affiliations:** Shanxi Key Laboratory of Animal Disease Research, Prevention and Control, College of Veterinary Medicine, Shanxi Agricultural University, Jinzhong 030801, China; liuxunzhi0206@126.com (X.-Z.L.); sunan1228@163.com (N.S.); 13934936109@163.com (W.L.); 17303450339@163.com (D.-Y.W.); zhangzedong0519@126.com (Z.-D.Z.); xingquanzhu1@hotmail.com (X.-Q.Z.)

**Keywords:** protozoan parasites, *Equus caballus*, molecular epidemiology, zoonotic transmission, North China

## Abstract

*Giardia duodenalis* (synonyms *G. lamblia* and *G. intestinalis*) and *Blastocystis* sp. are prevalent zoonotic intestinal protozoans with significant public health and economic importance. Both parasites infect various hosts, including humans and most livestock. *G. duodenalis* can cause infections ranging from asymptomatic carriage to clinical manifestations such as acute diarrheal disease, growth stunting and malabsorption, whereas *Blastocystsi* sp. infection is often asymptomatic and its pathogenicity remains debated. Following the rapid expansion of modern equine industries (horse racing, equestrian sports, production, and tourism), concern about the spread of parasitic diseases caused by human–horse contact has gradually increased. In this study, 631 horse fecal specimens were collected from three geographical localities in Shanxi Province, North China. The prevalence and genetic characteristics of the two parasites were determined by amplification of multiple genetic loci (*tpi*, triosephosphate isomerase; *gdh*, glutamate dehydrogenase; *bg*, beta-giardin) for *G. duodenalis* and the small subunit ribosomal RNA (SSU rRNA) gene for *Blastocystis* sp. Overall, the prevalence of *G. duodenalis* and *Blastocystis* sp. in horses in Shanxi Province was 7.9% and 0.8%, respectively. Sequence analysis identified three assemblages (A, B, and E) of *G. duodenalis* and two subtypes (ST1 and ST5) of *Blastocystis* sp. in horses; assemblage B and subtype ST1 were detected more frequently than the other types (subtypes) in this data set. This study is the first to report *G. duodenalis* and *Blastocystis* sp. infections in horses in Shanxi Province, and these findings provide baseline molecular epidemiological data for horses in Shanxi Province and support continued hygiene management to reduce potential zoonotic transmission.

## Simple Summary

Globally distributed, *Blastocystis* sp. and *Giardia duodenalis* represent two widespread enteric parasites capable of infecting both human and animal hosts, thereby raising public health concerns and veterinary significance. In horses, infection with these parasites may increase the risk of environmental contamination and potential zoonotic transmission through close contact between horses and humans. Shanxi Province is an important area for equine husbandry in North China. However, information on the prevalence of *Blastocystis* sp. and *G. duodenalis* in horses in Shanxi Province is still lacking. Therefore, 631 fecal samples from individual horses were collected from three representative localities of Shanxi Province to examine the occurrence and genetic characteristics of these two parasites using molecular methods. The results showed that the overall prevalence of *G. duodenalis* and *Blastocystis* sp. was 7.9% and 0.8%, respectively. Sequence analysis identified three assemblages of *G. duodenalis* and two subtypes of *Blastocystis* sp.; assemblage B and subtype 1 (ST1) were detected more frequently than the other types in this data set. This is the first report of *G. duodenalis* and *Blastocystis* sp. infections in horses in Shanxi Province, which provides baseline data for the prevention and control of these parasites in horses in the study area.

## 1. Introduction

*Giardia duodenalis* (Davaine, 1875) (synonyms *G. lamblia* and *G. intestinalis*) and *Blastocystis* sp. (Alexeieff, 1911) are prevalent intestinal protozoa, posing significant public health and economic burdens especially in low- and middle-income nations [1,2]. Clinical symptoms of *G. duodenalis* infection vary from asymptomatic carriage to acute diarrheal disease, growth stunting and malabsorption [3]. Approximately 300 million people, particularly children living in resource-limited settings, are affected annually worldwide. Although infections are often self-limiting in healthy individuals, prolonged infection and severe clinical outcomes may occasionally occur [4,5]. By contrast, *Blastocystis* infection is asymptomatic in most individuals, and, although some reports have linked *Blastocystis* with irritable bowel syndrome (IBS), its pathogenicity remains controversial [6,7,8].

Currently, the genus *Giardia* (Künstler, 1882) comprises nine taxonomically valid species, including *G. duodenalis*, *G. agilis*, *G. ardeae*, *G. psittaci*, *G. muris*, *G. microti*, *G. peramelis*, *G. cricetidarum* and *G. varani*, with *G. duodenalis* being the major species infecting domestic animals and humans [2,9]. *G. duodenalis* is considered a multispecies complex with eight genetic assemblages (A–H), which differ in host range, specificity and genetic traits [2]. Assemblages A and B are predominant assemblages detected in humans and most mammals, whereas assemblages C and D are mainly found in canids, E in hoofed animals, F in felids, G in rodents and H in marine pinnipeds [9,10]. A recent report showed that the prevalence of *G. duodenalis* in horses worldwide was 8.93%, with significant differences in prevalence among different countries and age groups [11]. Based on triosephosphate isomerase (*tpi*), glutamate dehydrogenase (*gdh*) and beta-giardin (*bg*) loci of *G. duodenalis*, assemblages A, B and E were detected in horses, with assemblage B as the predominant genotype, followed by assemblages A and E [11,12]. These findings suggest that horses may act as potential carriers or reservoirs of *G. duodenalis* and may contribute to transmission through direct contact or environmental contamination.

Based on polymorphisms in the small subunit ribosomal RNA gene (SSU rRNA) of *Blastocystis*, more than 40 subtypes of *Blastocystis* sp. have been identified in humans and animals (ST1–ST17, ST21, ST23–ST44), among which 12 subtypes (ST1–ST10, ST12 and ST14) have been recognized as zoonotic [13,14]. Globally, *Blastocystis* sp. was detected in horses in Egypt [15] and Iran [16], with prevalence rates of 3.3% and 11.5%, respectively. In China, no data are available on the prevalence of *Blastocystis* in horses, but a previous study in Shanxi Province showed that the prevalence of *Blastocystis* in donkeys, which belong to the genus *Equus* (Linnaeus, 1758), was 0.2% [17].

Shanxi Province, located in North China and characterized by a semi-arid continental monsoon climate, provides suitable conditions for equine husbandry through its temperate, seasonally humid–dry cycles [17]. Historically, as important working animals in China, horses were integral to transportation, agriculture and military operations. Nowadays, horses play increasingly important roles in modern equine industries, including horse racing and equestrian sports. Currently, increasing human–horse contact has raised concerns about horses serving as potential zoonotic reservoirs for the transmission of parasitic diseases to humans. Thus far, data on *G. duodenalis* in horses remain limited [12,18,19,20], and no data were available on *Blastocystis* infection among horses in China. Therefore, the present study employed a PCR-based molecular approach to determine the prevalence and genetic distribution of both parasites in horses in Shanxi Province, North China.

## 2. Materials and Methods

### 2.1. Sampling Collection

Following the reported prevalence of *G. duodenalis* in horses and *Blastocystis* sp. in horses/donkey in China, the minimum representative sample sizes required for this study were estimated to be 64 and 9 using the Thrusfield’s [21] formula *n* = [1.962 × P_exp_(1 − P_exp_)]/d^2^ (*n* = the required sample size; P_exp_ = expected prevalence; d = desired absolute precision).

**Figure 1:**
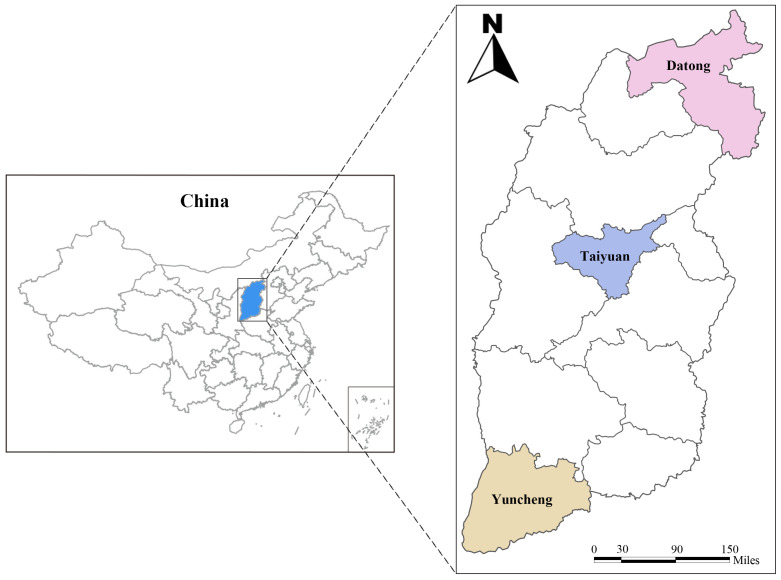
Sampling sites of horse specimens in this study.

From March to June 2023, a total of 631 fresh fecal specimens were collected from three separate localities located in the northern (Datong City; *n* = 269), central (Taiyuan City; *n* = 261), and southern (Yuncheng City; *n* = 101) parts of Shanxi Province, North China (Figure 1). Each fecal specimen represented one host animal. Samples were collected from freshly voided feces; the top layer was collected immediately and deposited into sterile polyethylene (PE) gloves to minimize contamination from the portion in contact with the ground. At the same time, detailed information, including geographic location, age, gender, and management system was recorded. The 631 samples were classified into two age groups (≥3 years and <3 years), three gender groups (male, female, and gelding), and two management systems (racehorses and farm horses). All sampled horses were apparently healthy based on farm records and visual inspection, with no obvious diarrhea at sampling. The collected samples were preserved in 2.5% potassium dichromate at 4 °C and transported to the Laboratory of Parasitic Diseases, College of Veterinary Medicine, Shanxi Agricultural University.

### 2.2. DNA Extraction and PCR Amplification

Prior to DNA extraction, every fecal sample was washed three times with distilled water to discard potassium dichromate. Subsequently, genomic DNA was extracted from each sample using the E.Z.N.A.^®^ Stool DNA Kit (Omega Bio-Tek Inc., Norcross, GA, USA) as instructed by the manufacturer, and stored at −20 °C before PCR amplification. The primers and protocols for PCR amplification and fragment analysis were as previously described [17,22]. Briefly, the prevalence of the two parasites was examined by PCR amplification targeting the *tpi*, *gdh* and *bg* loci of *G. duodenalis* and SSU rRNA of *Blastocystis* sp., respectively. A total of 25 µL PCR mixture consisted of 2.5 µL of 10× PCR Buffer (Mg^2+^ free), 2 µL of dNTPs Mix, 2 mM of MgCl_2_ (25 mM), 1.25 U of *Ex*-Taq DNA polymerase (Takara, Dalian, China), 0.25 μM of each primer, 2 µL of DNA template, and 14.5 µL of double-distilled water (ddH_2_O). Positive controls consisting of *G. duodenalis* or *Blastocystis* DNA extracted from horses and a negative control (ddH_2_O) were included in each round of amplification to ensure the accuracy and reliability of the amplification results. PCR products were visualized by electrophoresis on 1.5% agarose gels containing ethidium bromide (EB) under UV light, and positive products were sent to Sangon Biotech Co., Ltd. (Shanghai, China) for bidirectional sequencing.

### 2.3. Sequencing and Phylogenetic Analysis

After aligning and assembling the bidirectional sequencing chromatograms using ChromasPro v2.1.3, the obtained sequences were used for further analysis. Using the Basic Local Alignment Search Tool (BLASTn, NCBI web server), raw sequences were matched with reference sequences stored in the NCBI database for genotyping and subtyping of the two parasites. Subsequently, identified sequences were used to construct a phylogenetic tree using the Neighbor-Joining (NJ) method and the Kimura 2-parameter model in MEGA7.0 software to assess genetic relationships between known genotypes or subtypes and the sequences of *G. duodenalis* or *Blastocystis* sp. identified in the present study [22]. The reliability of the phylogenetic tree was verified using a bootstrap analysis with 1000 replicates.

### 2.4. Statistical Analysis

The associations between the prevalence of each parasite and risk factors (localities, age, gender and management pattern) were analyzed using SPSS 26.0 software (SPSS Inc., Chicago, IL, USA) using chi-square (χ^2^) test. Likewise, odds ratios (ORs) and their 95% confidence intervals (95% CI) were used to assess the associations between prevalence and risk factors. Differences were considered statistically significant when the *p*-value was less than 0.05.

## 3. Results

### 3.1. Prevalence of G. duodenalis and Blastocystis *sp.* in Horses in Shanxi Province

Based on the *bg*, *tpi*, and *gdh* loci, 44, and 15 out of 631 fecal samples tested positive for *G. duodenalis* at the *bg* and *tpi* loci, respectively, whereas no positive sample was detected at the *gdh* loci with an overall prevalence of 7.9% (50/631). As shown in Table 1, the highest prevalence of *G. duodenalis* was detected in Taiyuan City (15.3%), followed by Yuncheng City (5.0%) and Datong City (1.9%), with a significant difference among the three sampling localities (*p* < 0.001). Between age groups, no statistically significant difference was observed in prevalence, with prevalence of 8.2% and 6.6% in horses older than 3 years and less than 3 years, respectively. The prevalence of *G. duodenalis* differed among the three gender groups, ranging from 6.3% in female horses (31/494) to 18.8% in gelding horses (6/32). Notably, the prevalence of *G. duodenalis* in horses from racecourses was 15.3% (40/261), which was significantly higher than that in farm horses with a prevalence of 2.7% (10/370) (*p* < 0.001).

Of the 631 fecal samples collected from horses in Shanxi Province, five (0.8%) samples tested positive for *Blastocystis* sp. (Table 1). Among the three sampling localities, the prevalence of *Blastocystis* was higher in Taiyuan City (1.2%, 3/261) than in Yuncheng City (1.0%, 1/101) and Datong City (0.4%, 1/269). Between age groups, the prevalence of *Blastocystis* in horses less than 3 years old (2.2%, 2/91) was slightly higher than that in horses older than 3 years (0.6%, 3/540). However, no statistically significant associations were observed between prevalence and the associated factors, including sampling localities (*p* = 0.583), age group (*p* = 0.102), and management group (*p* = 0.396). Across different gender groups, all five positive samples were detected in female horses, but no significant difference was detected among gender groups (*p* = 0.497).

### 3.2. Genotype and Sub-Genotype Identification of G. duodenalis in Horses

To confirm the genetic population of *G. duodenalis* in this study, the obtained sequences were genotyped by BLAST analysis, and the results are shown in Table 2. The nucleotide sequences of *G. duodenalis* obtained in this study were submitted to the GenBank database under accession numbers PV844244 to PV844254 (*bg*) and PV844255 to PV844257 (*tpi*). Based on the *bg* locus, 44 sequences were assigned to assemblage B (*n* = 40), followed by assemblage A (*n* = 3) and E (*n* = 1), with assemblage B as the main detected genotype in horses in Shanxi Province. At the *tpi* locus, 15 sequences were obtained, including 13 assemblage B sequences and two assemblage A sequences, indicating that assemblage B was the most prevalent genotype in fecal samples from horses. However, no positive samples were identified at the *gdh* locus, and assemblage assignment was therefore based on the *bg* and *tpi* results rather than complete multi-locus sequence typing. Interestingly, the positive samples collected in Datong City and Yuncheng City were amplified only at the *bg* locus, and all sequences were identified as assemblage B. In total, assemblage A and E sequences identified in this study were only detected in horses from Taiyuan City. In addition, assemblages A, B and E were detected in horses older than 3 years, whereas only assemblage B was detected in horses less than 3 years. Across the gender groups, both assemblages A and B were detected in male horses, and assemblages A, B and E were detected in female horses, but only assemblage B was detected in gelding horses. Similarly, assemblage B was the only genotype detected in farm horses, whereas assemblages A, B, and E were detected in racehorses.

**Figure 2:**
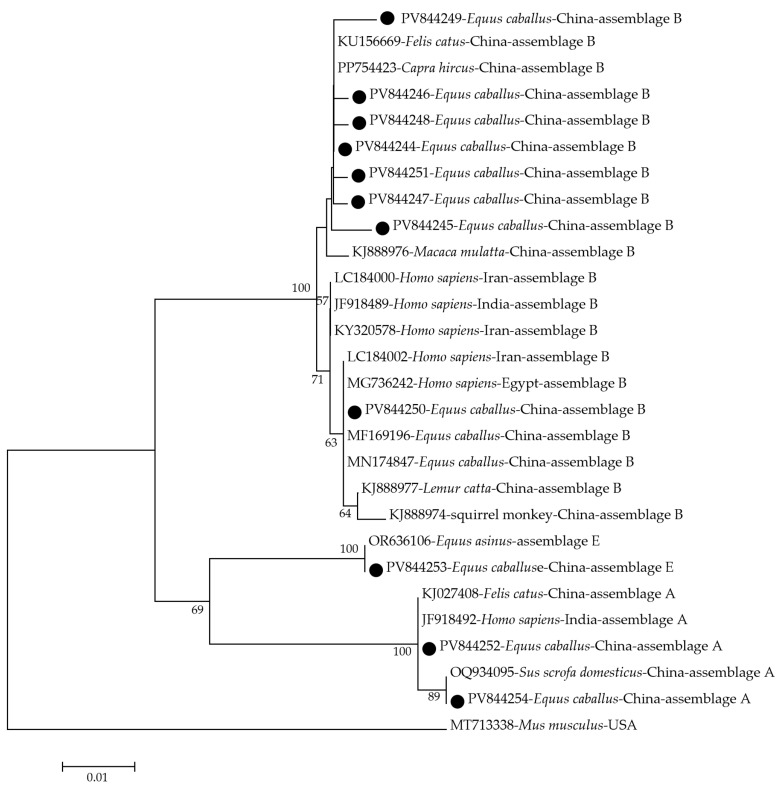
Phylogenetic tree of *G. duodenalis* β-giardin (*bg*) sequences constructed using the Neighbor-Joining method. Isolates obtained in this study were indicated by black circles.

At the *bg* locus, further sequence analysis showed that 40 out of 44 sequences belonged to assemblage BIII. Analysis of single nucleotide polymorphisms (SNPs) in assemblage B showed that 75.0% (30/40) of the sequences were 99.42–100.00% similar to the reported BIII subgroup from black goats in Shanxi Province (PP754423), whereas the remaining 10 sequences shared 99.81–100.00% similarity with the reference sequence from *Homo sapiens* in Egypt (MG736242) (Table 3). Three obtained assemblage A sequences were identified as sub-assemblage AI (*n* = 2) and AII (*n* = 1), showing 100% similarity to a reported sub-assemblage AI sequence from Tan sheep in Ningxia Province of China (MK610391) and AII sequence from cat in Guangdong Province of China (KJ027408), respectively. One assemblage E sequence identified at the *bg* locus showed 100% similarity to a reported assemblage E sequence from donkeys in the Shanxi Province of China (OR636106). In addition, a phylogenetic tree based on the *bg* locus was constructed to assess the genetic distance of identified sequences with known assemblages (Figure 2). Phylogenetically, the sequences belonging to each assemblage (A, B, and E) clustered into a single clade, and each clade contained multiple sequences identified from humans and other livestock.

At the *tpi* locus, 15 sequences were identified, including 13 assemblage B sequences and two assemblage A sequences. Among the 13 assemblage B sequences, 12 sequences and one other sequence showed 100.00% and 99.81% similarity, respectively, to a known sub-assemblage B sequence (OQ947879) previously reported in donkeys in Shanxi Province. Two assemblage A sequences shared 100.00% identity with the reported sub-assemblage AII sequence from *Homo sapiens* in Iran (LC183952).

### 3.3. Subtypes (ST) Identification of Blastocystis in Horses

Among the five *Blastocystis*-positive samples identified in this study, three sequences were identified as ST1 and the other two as ST5. Two of the ST1 sequences shared 100% identity with a known ST1 sequence (PQ877672) previously reported in *Microtus* from Henan Province, China, while the remaining ST1 sequence showed complete identity (100%) to another known ST1 sequence (ON062437) reported in beef cattle from Shanxi Province, China. Both sequences identified as ST5 exhibited 100% similarity to a known ST5 sequence (OM859026) identified in pigs from Shanxi Province, China. Three representative sequences obtained in this study were deposited in the GenBank database under accession numbers PX426274 to PX426276.

## 4. Discussion

*G. duodenalis* and *Blastocystis* are prevalent zoonotic intestinal protozoans with significant public health and economic importance [3,8]. Following infection, both parasites colonize the host intestine and shed millions of cysts into the environment, thereby maintaining environmental contamination and serving as potential sources of infection for humans or other animals [9,23,24]. Previous reports have estimated that approximately 10% of the world’s population was infected with *G. duodenalis*, whereas more than one billion people were infected with *Blastocystis* [4,25]. In China, horses are important working and racing animals; however, only a few studies have investigated the prevalence of these parasites in horses in China, and no data were available for horses in Shanxi Province [12,18,19,20]. Therefore, the present study used molecular methods to determine the occurrence of the two parasites in horses in Shanxi Province, North China, and identified prevalences of 7.9% for *G. duodenalis* and 0.8% for *Blastocystis*.

Globally, a report indicated that the overall prevalence of *G. duodenalis* in horses ranged from 0% to 12.2%, with the highest prevalence detected in Europe (12.2%), followed by Asia (11.8%), and the Americas (11.82%) [11]. In the present study, *G. duodenalis* was detected more frequently than *Blastocystis* sp. among the two parasites examined in 631 horses sampled in Shanxi Province, with an overall prevalence of 7.9%. This prevalence was higher than those reported in Nigeria (0%) [26], Brazil (0.5%) [27], Australia (2.3%) [28], Canada (5.8%) [29] and Portugal (7.6%) [30], but lower than those reported in Mexico (72.7%) [31], Iran (35.7%) [32] and Turkey (16.7%) [33]. In China, previous studies have reported that the prevalence of *G. duodenalis* in horses ranges from 1.5% to 9.4%, with the highest prevalence reported in the Qinghai–Tibetan Plateau Area [20] and the lowest in Xinjiang [19]. A previous study demonstrated that geographical differences in prevalence may be related to horse breed, breeding conditions, local climatic conditions, sampling season and diagnostic method [11]. More detailed epidemiological information can help researchers to understand the associations between these factors and the prevalence of *G. duodenalis* in horses.

Notably, the present study revealed significant regional variation in prevalence within Shanxi Province. The prevalence of *G. duodenalis* in Taiyuan City (15.3%) was markedly higher than that in Datong City (1.9%) and Yuncheng City (5.0%). This difference may be attributable to Taiyuan’s status as the provincial capital of Shanxi Province, which is characterized by high population density and substantial traffic flow and serves as a major transit hub for railways and highways connecting neighboring provinces. High mobility of people and animals facilitates the dissemination of parasites, including *G. duodenalis*, and may increase the risk of bidirectional transmission between humans and animals. As a globally distributed parasite, the recognized association between *G. duodenalis* infection and travel-related diarrhea further underscores the potential role of host mobility in parasite spread [34].

Between the two age groups, the current study showed that the prevalence of *G. duodenalis* in older horses (8.2%) was slightly higher than that in younger horses (6.6%), but the difference was not statistically significant (*p* = 0.611). In contrast, a recent meta-analysis indicated that horses younger than 3 years were more susceptible to *G. duodenalis* than horses older than 3 years, and the prevalence differed significantly between age groups [11]. Younger animals are generally considered more susceptible to giardiosis due to their immature immune systems [2]. In the present study, however, only 91 fecal samples were collected from horses younger than three years of age, which may have limited the statistical power to detect age-related differences. Additional samples should be collected in subsequent studies to more accurately assess the relationship between age and *G. duodenalis* in horses.

Our analysis also revealed significant differences in prevalence among the three gender groups and between management patterns. The prevalence in gelding horses (18.8%) was significantly higher than that in male horses (12.4%) and female horses (6.3%) (*p* = 0.007). While our results cannot directly establish causality, previous research has suggested that gelding horses exhibit distinct immune and inflammatory regulation patterns, including heightened immune activation and oxidative stress responses [35]. Prolonged inflammatory states may impair immune regulation, and reduced testosterone levels following castration could potentially compromise mucosal immunity, creating a more permissive environment for parasite colonization [35]. Further studies are needed in the future to provide evidence verifying the correlation between gelding status and parasitic infection. Management pattern was another factor that significantly influenced the prevalence of *G. duodenalis* in horses. In this study, horses reared on farms exhibited a lower prevalence (2.7%) compared to horses from racecourses (15.3%). This difference may reflect increased *G. duodenalis* transmission opportunities with higher human traffic and animal density at racecourses.

Numerous studies pointed out that assemblage B is the most common *G. duodenalis* genotype in horses both in China and worldwide [20,36,37]. Consistently, assemblage B was the most prevalent genotype detected in horses in the present study, followed by assemblages A and E. Assemblages A and B have been widely reported in both humans and animals and are responsible for most human infections, whereas assemblage E is usually detected in hoofed animals [10]. In this study, two sequences were identified as sub-assemblage AI at the *bg* locus; in addition, one sequence at the *bg* locus and one sequence at the *tpi* locus were identified as sub-assemblage AII. Previous studies indicated that sub-assemblage AI was specific to humans and AII was considered a zoonotic sub-genotype, whereas AIII and AIV were reported in animals [38,39]. Regarding assemblage B, four sub-assemblages (BI–BIV) of assemblage B have been identified, with BIII as the zoonotic genotype [39]. The presence of AI, AII and BIII in horses in this study indicated that horses may be potential carriers of *G. duodenalis* and could contribute to environmental contamination or zoonotic transmission by direct or indirect contact. Unfortunately, no positive amplification was detected at the *gdh* locus in this study, likely due to primer–template mismatch, PCR amplification efficiency or DNA degradation.

In this study, five (0.8%) out of 631 samples tested positive for *Blastocystis*, suggesting that horses in Shanxi Province may not be highly susceptible to *Blastocystis* infection. Statistical analysis revealed no significant association between *Blastocystis* prevalence and the investigated factors, including study localities, age, gender and management pattern (*p* > 0.05). By contrast, previous studies in Shanxi Province showed that the prevalence of *Blastocystis* was significantly related to the study localities in sheep [22] and donkeys [17]. In addition, sequence analysis further identified two *Blastocystis* subtypes, ST1 and ST5, among the five positive samples. Increasing evidence suggests that ST1 has strong pathogenic potential and is associated with humans and various animals [40,41], indicating its zoonotic transmission potential at the human–horse contact.

## 5. Conclusions

The present study demonstrated the occurrence and genetic diversity of *G. duodenalis* and *Blastocystis* sp. in horses in Shanxi Province, North China, with overall prevalence rates of 7.9% for *G. duodenalis* and 0.8% for *Blastocystis*. Sequence analysis revealed three assemblages (A, B, and E) of *G. duodenalis* and two subtypes (ST1 and ST5) of *Blastocystis* in the study localities, including the zoonotic sub-genotypes AI, AII, and BIII of *G. duodenalis* and the zoonotic subtype ST1 of *Blastocystis*. Our results indicate that horses may serve as potential sources for transmission of both parasites. Thus, stricter hygiene management, routine surveillance and measures to reduce direct and indirect transmission should be taken to minimize the risk of zoonotic parasitic disease transmission to humans and other animals.

## Figures and Tables

**Table 1:** Factors associated with the prevalence of *Giardia duodenalis* and *Blastocystis* sp. in horses in Shanxi Province.

Parasites	Parameters	Factors	No. Positive/Tested	Prevalence % (95% CI)	OR (95% CI)	*p*-Value
*Giardia duodenalis*	localities	Datong	5/269	1.9 (0.2–3.5)	1	<0.001
		Taiyuan	40/261	15.3 (11.0–19.7)	9.6 (3.7–24.6)	
		Yuncheng	5/101	5.0 (0.7–9.2)	2.8 (0.8–9.7)	
	Age	≥3 years	44/540	8.2 (5.8–10.5)	1.3 (0.5–3.0)	0.611
		<3 years	6/91	6.6 (1.5–11.7)	1	
	Gender	Male	13/105	12.4 (6.1–18.7)	2.1 (1.1–4.2)	0.007
		Female	31/494	6.3 (4.1–8.4)	1	
		Gelding	6/32	18.8 (5.2–32.3)	3.4 (1.3–9.0)	
	Management	Racehorses	40/261	15.3 (11.0–19.7)	6.6 (3.2–13.3)	<0.001
		Farm horses	10/370	2.7 (1.1–4.4)	1	
	Total		50/631	7.9 (5.8–10.0)		
*Blastocystis* sp.	localities	Datong	1/269	0.4 (0–1.1)	1	0.583
		Taiyuan	3/261	1.2 (0–2.4)	3.1 (0.3–30.4)	
		Yuncheng	1/101	1.0 (0–2.9)	2.7 (0.2–43.3)	
	Age	≥3 years	3/540	0.6 (0–1.2)	1	0.102
		<3 years	2/91	2.2 (0–5.2)	4.0 (0.7–24.4)	
	Gender	Male	0/105	0	1	0.497
		Female	5/494	1.0 (0.1–1.9)	1.0 (1.0–1.0)	
		Gelding	0/32	0	1	
	Management	Racehorses	3/261	1.2 (0–2.4)	2.1 (0.4–12.9)	0.396
		Farm horses	2/370	0.5 (0–1.3)	1	
	Total		5/631	0.8 (0.1–1.5)		

**Table 2:** Distribution of *Giardia duodenalis* assemblages in horses in Shanxi Province according to localities, age, gender, and feeding pattern.

Factor	Category	Case Counts	No. of Positive	Assemblage A (*n*)			Assemblage B (*n*)			Assemblage E (*n*)		
				*bg*	*tpi*	*gdh*	*bg*	*tpi*	*gdh*	*bg*	*tpi*	*gdh*
localities	Datong	269	5	0	0	0	5	0	0	0	0	0
	Taiyuan	261	40	3	2	0	30	13	0	1	0	0
	Yuncheng	101	5	0	0	0	5	0	0	0	0	0
Age	≥3 years	540	44	3	2	0	35	11	0	1	0	0
	<3 years	91	6	0	0	0	5	2	0	0	0	0
Gender	Male	105	13	1	1	0	11	3	0	0	0	0
	Female	494	31	2	1	0	24	7	0	1	0	0
	Gelding	32	6	0	0	0	5	3	0	0	0	0
Management	Racehorses	261	40	3	2	0	30	13	0	1	0	0
	Farm horses	370	10	0	0	0	10	0	0	0	0	0
	Total	631	50	3	2	0	40	13	0	1	0	0

**Table 3:** Analysis of single nucleotide polymorphisms in *G. duodenalis* sequences at *bg* locus.

Sequences	Nucleotide at Position of Reference Sequence															No. of Sequences
Assemblage B	0	1	54	76	133	144	177	202	219	250	259	299	383	423	426	
PP754423-BIII	G	A	T	C	C	A	A	G	T	C	C	A	C	A	T	
PV844244	G	A														25
PV844245	G	A				T				G		T				1
PV844246	G	A													C	1
PV844247	G	A							C							1
PV844248	G	A					G									1
PV844249	G	A	C		T									G		1
PV844250	G	A		T				A					T			9
PV844251	G	A									T					1
Assemblage A	0	1	67	317												
JF918492	G	A	C	C												
PV844252	G	A														1
PV844254	G	A	T	T												2
Assemblage E	0	1														
OR636106	G	A														
PV844253	G	A														1

## Data Availability

The data sets supporting the results of this article have been submitted to GenBank, and the accession numbers are shown in the article.
